# Host specific endophytic microbiome diversity and associated functions in three varieties of scented black rice are dependent on growth stage

**DOI:** 10.1038/s41598-021-91452-4

**Published:** 2021-06-10

**Authors:** K. Malabika Singha, Brahmanand Singh, Piyush Pandey

**Affiliations:** 1 Department of Microbiology, Assam University, Silchar, 788011 India; 2 Department of Pharmacognosy and Ethnopharmacology, CSIR-National Botanical Research Institute, Lucknow, Uttar Pradesh 226001 India

**Keywords:** Microbiology, Plant sciences

## Abstract

The compositional and functional role of the endophytic bacterial community, associated with black scented rice, in correlation with its antioxidant property has been elucidated. Community dissimilarity analysis confirmed the overlapping of community in shoot and root tissues at the young stage, but not in mature plants. Proteobacteria was the most abundant phylum, in which *Agrobacterium, Pleomorphomonas, Bradyrhizobium, Novasphingobium, Caulobacter* were the most abundant genera, followed by Cyanobacteria and Planctomycetes in all three different varieties of the black rice. The antioxidant activity of mature plants was found to be higher in comparison to young plants. Intrinsically, the relative abundance of *Pleomorphomonas* and *Streptomyces* was positively correlated with total phenol content, while *Gemmata*, *unclassified Pirellulaceae*, *unclassified Stramenopiles* positively correlated with total flavonoid content and negatively correlated with Free radical scavenging activity. Accordingly, functional metagenome analysis of the endophytic microbiome revealed that naringenin -3-dioxygenase and anthocyanidin 3-O-glucosyltransferase for phenylpropanoid (flavonoid and anthocyanin) synthesis were abundant in the endophytic microbiome of mature plants. Specific enrichment of the antioxidant producing genes in the mature plant endophytic microbiome was assigned to some bacteria such as *Streptomyces, Pantoea* which might have contributed to the common pathway of flavonoid synthesis*.* The genomes of endophytic isolates *Kluyvera* sp*.*PO2S7, *Bacillus subtilis* AMR1 and *Enterobacter sp.* SES19 were sequenced and annotated, and were found to have genes for phenylpropanoid synthesis in their genomes.

## Introduction

Plant microbiomes are important to host adaptation and impact plant productivity, health^1^, and known to affect plant traits as well as disease resistance, growth, and abiotic stress tolerance^2^. The various studies have established that endophytic bacterial communities change with the varieties and age of the host. As genetic adaptation is comparatively slow in plants, the plant microbiome helps more quickly to adjust to a changing environment^3^. The endophytic microbiome assemblage in plants depends on two factors, overall selection from the neighboring area of the root, and species-specific genetic factors that facilitate entrance inside the root^4^. The microbiota colonizing the endophytic compartment helps in the differentiation of microbial community across plant species^5^.

The plant endophytic microbiome investigation enables connecting microbial ecology, with host plant biology and observe microorganisms as a reservoir of additional genes and functions for their host^6^. By using metagenomic methods, uncultured microbial communities are analyzed and the whole microbiome is being studied for gene content to identify the interaction of the endophytic community by its protein-coding genes^7^. Also, another understudied probable source of microbiome variation is host-age, which can affect the expression of plant functional characters that impact the microbiome^8^. However, little work has been done to connect these functions or putative beneficial traits with the endophytic microbiome.

There are several reports on endophytic bacterial diversity in rice plants, which have been assessed using 16S rRNA-based techniques^9^, however, the endophytic bacterial community of black-rice has not been characterized yet. Therefore, in the present study, microbiomes associated with the shoots and roots of three varieties of scented black rice (*Amubi, Poreiton*, and *Sempak*) have been studied in different stages of plant growth along with its correlation between taxonomic composition and the functional attributes.

Black scented rice, Chakhao (*Oryza sativa* L.) (Supplementary Fig. S1) is important for its unique fragrance, high antioxidant (phenolic), anthocyanin content, and helpful for cardiovascular health^10^. Better antioxidant activities in a plant are seen as an attribute for its commercial values and are also important for the plants, as it helps in reducing many abiotic stresses in the plant tissues^11^. It has been reported that the inoculation of endophytes induces the synthesis of phenylpropanoid (antioxidant) in plants^12^. Endophytes synthesize secondary metabolites via a variety of pathways, e.g., polyketide, isoprenoid, or amino acid derivation^13^. Interestingly, numerous studies report the bioactive metabolites that are produced either by endophytes or their host plants^14^. The endophytes dwelling in such chemically bioactive niche within plant materials would indicate sharing biosynthetic genes for bioactive metabolites.

Although the promotion of the growth of black rice by plant growth-promoting probiotic bacteria have been described in several reports^15^, no information is available on the influence of the endophytic microbiome, and its variation regarding the stage of growth, and content of antioxidants. Therefore, the diversity of endophytic bacteria with the potential for antioxidant associated with black rice remains to be characterized in terms of plant age and stage of development.

However, there has been limited progress in understanding how the functional capabilities of endophytic bacterial communities change across plant development. It was hypothesized that the composition and diversity of endophytic bacteria could be different among different genotypes of black rice. Also, as this plant is known for its antioxidant content, it was interesting to correlate the endophytic microbiome with antioxidant activity. Here, we aimed to investigate the changes in total flavonoid content, antioxidant capacity, and phenolic acid composition of *Amubi, Poreiton,* and *Sempak* varieties of black rice at young and mature stages, along with functional analysis of the endophytic microbiome, as determined to establish linkage between key bacterial taxa, antioxidant activity, and the role of the microbiome (if any). So, the main objective of the study is to analyze the endophytic bacterial community of black rice at two distinct physiological stages of development: the young and mature stage in the root and shoot of three varieties of scented black rice (*Amubi, Poreiton, and Sempak*) and their correlation with antioxidant activity.

## Results

### Dissimilarities in the endophytic bacterial community in roots and shoots of three varieties of the scented black rice plant were influenced by plant development

Shannon and Simpson's indices were higher in root compared to the corresponding shoot except in *Poreiton* variety. Among all the samples, the *Amubi* root had the highest Simpson (0.9392) and Shannon (3.766) index while the *Sempak* root had the lowest Shannon (0.2943) and Simpson (0.7532) indices respectively (Supplementary Table 2). The rarefaction curves attained from the complete data set were comparable and given in supplementary Fig. S2.

**Figure 1:**
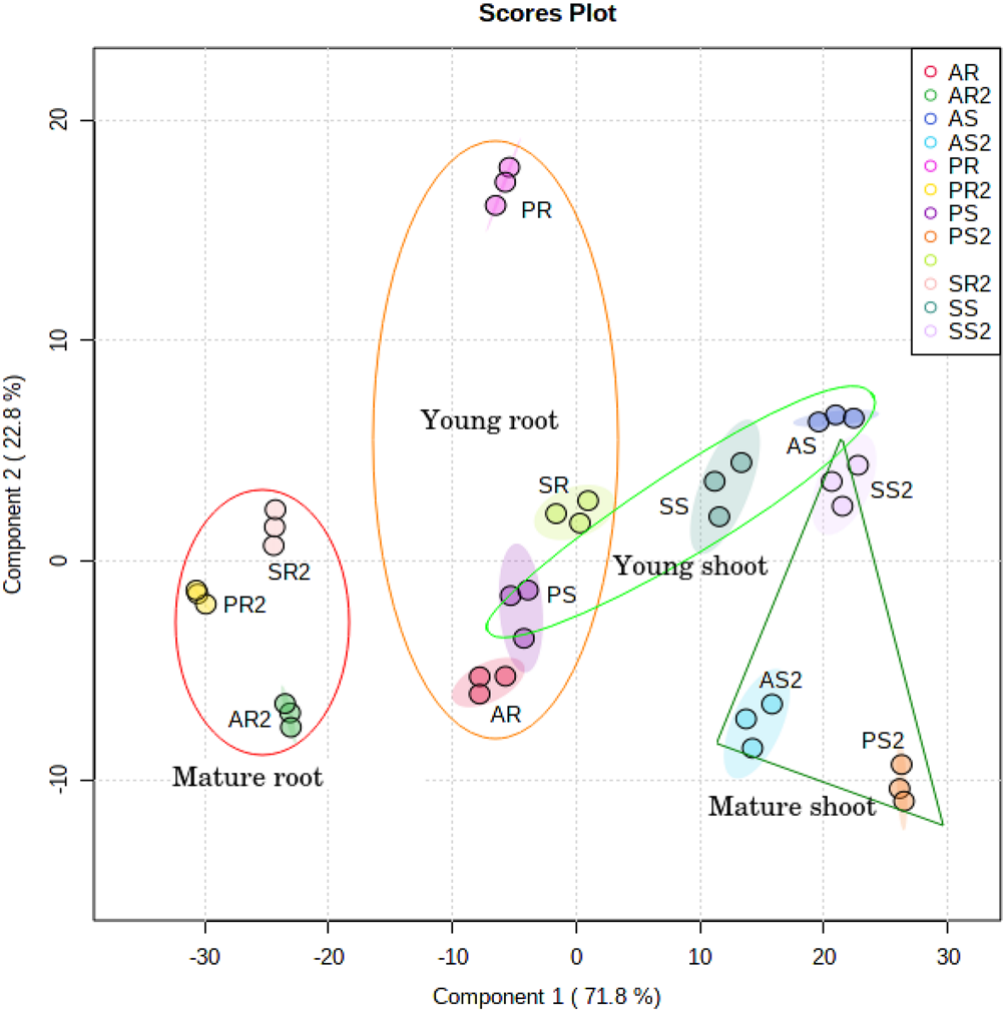
Multivariate analysis of the endosphere microbial community through plant development analysed by MiSeq sequencing. Principal Coordinate Analysis (PCoA) for the visualization of pairwise community dissimilarity (Bray–Curtis dissimilarity) of the endosphere microbial community at young and mature plant developmental stage. 95% confidence ellipses and triangles are shown around each developmental stage and plant part. [AR-Young *Amubi* root, AR2-Mature *Amubi* root; AS-Young *Amubi* shoot, AS2-Mature *Amubi* shoot; PR-Young *Poreiton* root, PR2-Mature *Poreiton* root, PS-Young *Poreiton* shoot, PS2-Mature *Poreiton* shoot; SR-Young *Sempak* root, SR2-Mature *Sempak* root , SS-Young *Sempak* shoot, SS2-Mature *Sempak* shoot].

The principal coordinate analysis (PCoAs) of Bray–Curtis distances was performed to determine the dissimilarity of the endophytic microbial communities between young and mature stages of the plant was described by the first coordinate (PCoA1) that described 71.8% of the variance, and the second coordinate (PCoA2) described 22.8% of the variance (Fig. 1). The statistically (*P* < 0.05) disperse endophytic bacterial community was observed in mature roots and shoots, from that of young plants (young root, young shoot, Fig. 1). The data from PERMANOVA and ANOSIM analysis suggested that the separate clusters as obtained for each sample, derived either from young root, young shoot, mature root, or mature shoot, were significantly distinct for all the three varieties (one-way PERMANOVA, *P* = 0.0001; ANOSIM values of R = 0.7037, *P* = 0.0001). However, the young root and young shoot reserved a similar endophytic microbial community (Fig. 1). As the plant developed, there were significant increase in the variation of endophytic bacterial communities in shoot and root of each respective varieties in comparison to young root and shoot (one-way PERMANOVA, *P* = 0.0002; ANOSIM values of R = 0.0815, *P* = 0.0001) (Fig. 1).

**Figure 2:**
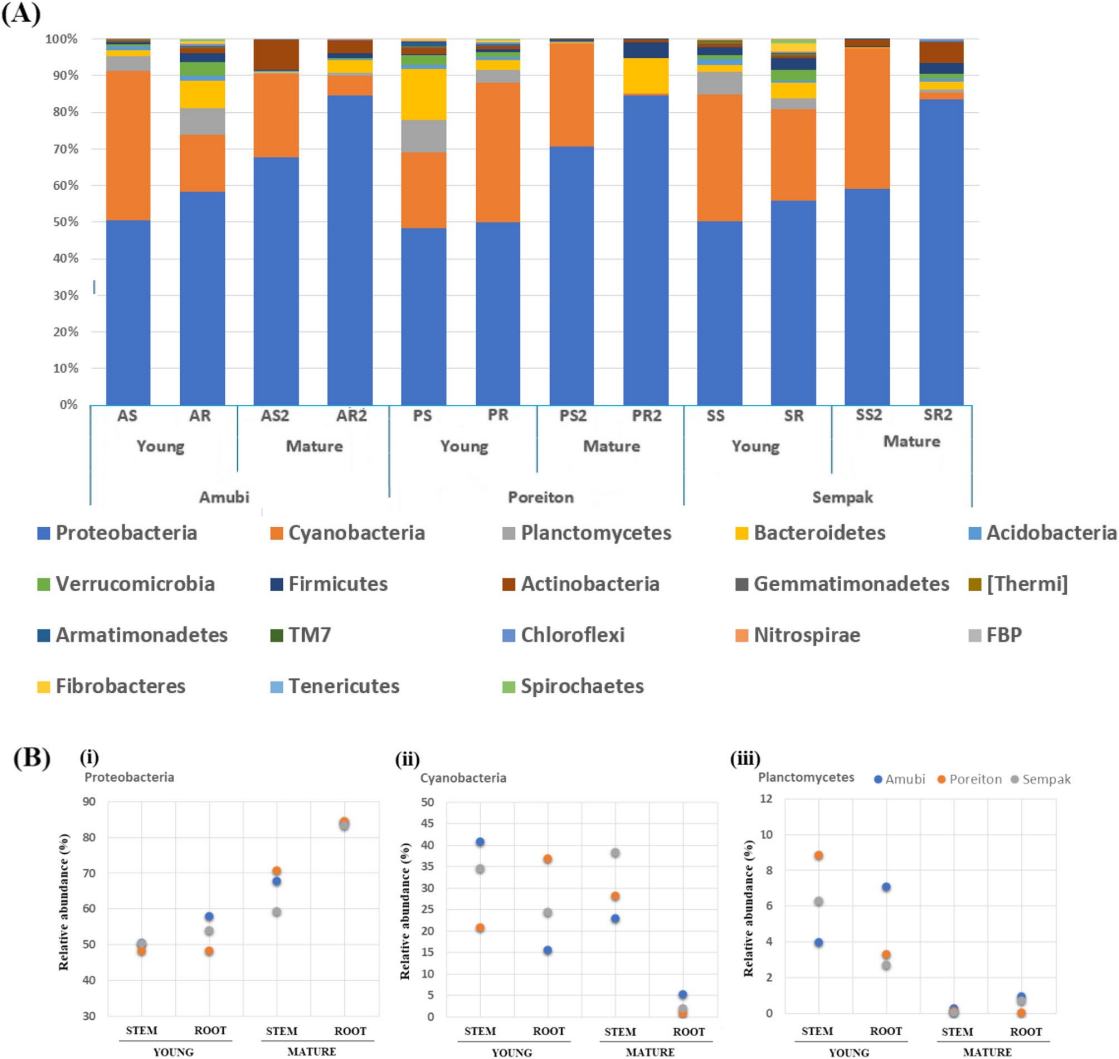
(**A**) Relative abundance (%) of the major bacterial phyla present in the endosphere microbial community at two developmental stage in three variety. [AR-Young *Amubi* root, AR2-Mature *Amubi* root; AS-Young *Amubi* shoot, AS2-Mature *Amubi* shoot; PR- Young *Poreiton* root, PR2-Mature *Poreiton* root, PS-Young *Poreiton* shoot, PS2-Mature *Poreiton* shoot; SR-Young *Sempak* root, SR2-Mature *Sempak* root, SS-Young *Sempak* shoot, SS2- Mature *Sempak* shoot]. (**B**) Bacterial phyla that significantly change with plant development in 3 varieties (*Amubi, Poreiton* and *Sempak*) of black rice (i) Proteobacteria, (ii) Cyanobacteria, (iii) Planctomycetes.

From the whole metagenome analysis, it was observed that most of the endophytic microbiome of black rice was comprised of bacteria, and the abundance of fungi, other microbes were found to be very less in both young and mature stages as given in Krona graph (Supplementary Figs. S3 and S4). The endophytic bacterial community was classified into phylotypes, which were consisted of 30 phyla (Fig. 2A), where Proteobacteria was the most abundant phyla irrespective of the varieties, and root or shoot tissues, accounting for > 80% in roots and > 60% in shoots, respectively. Taxonomy analysis identified significant differences in abundance at different developmental stages in root and shoot of phylum Proteobacteria, Cyanobacteria, and Planctomycetes mainly (Fig. 2B). The relative abundance of Proteobacteria significantly increased from young to mature plants in both roots (48.13–84.44%) and shoot (48.32–70.71%) and its abundance were more in root compared to shoot. On the contrary, the abundance of Cyanobacteria decreased in the mature root of all varieties while in shoot it remained consistent. Planctomycetes were present only in young root and shoot and its abundance decreased drastically in mature plants in all three varieties (8.85–0.01%). Similarly, we detected an increase in the abundance of Bacteroidetes from shoot to root except in *the Poreiton* stem.

**Figure 3:**
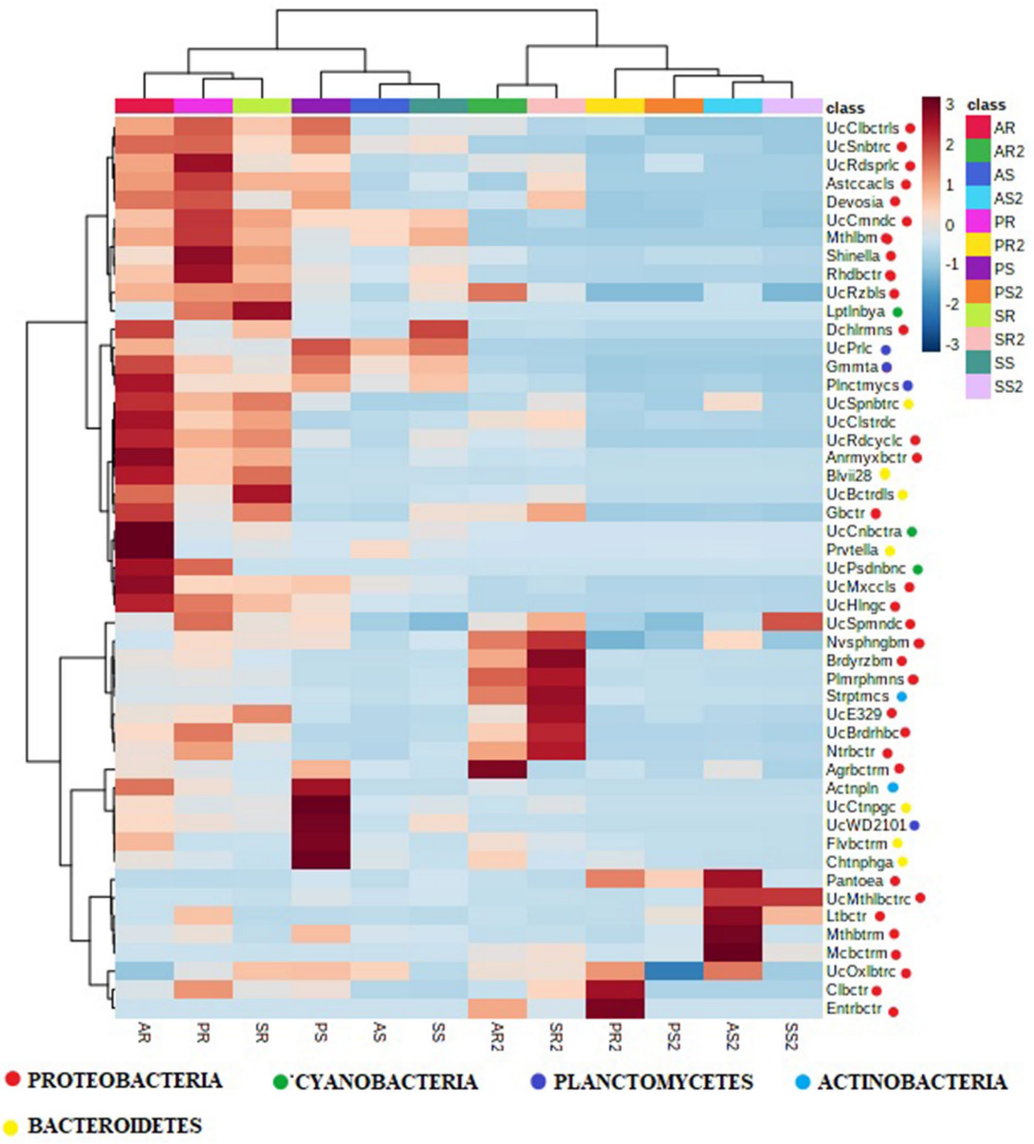
Heatmap of log absolute abundance of top 50 genera ranked by *t*-tests to retain the most contrasting patterns present in the endosphere microbial community of root and shoot at two developmental stage in three varieties (*Amubi, Poreiton* and *Sempak*) of black rice. Log absolute abundance of the microbes were scaled in a range of − 3 to 3 and clustered according to Euclidian distance matrix. [AR-Young *Amubi* root, AR2-Mature *Amubi* root; AS-Young *Amubi* shoot, AS2-Mature *Amubi* shoot; PR-Young *Poreiton* root, PR2-Mature *Poreiton* root, PS-Young *Poreiton* shoot, PS2-Mature *Poreiton* shoot; SR-Young *Sempak* root, SR2-Mature *Sempak* root, SS-Young *Sempak* shoot, SS2-Mature *Sempak* shoot].

Additionally, the genera, whose abundance increased in mature root compared to young root were *Enterobacter* (Entrbctr), *Caulobacter* (Clbctr)*, Pleomorphomonas* (Plmrphmns)*, Agrobacterium* (Agrbctrm)*, Novasphingobium* (Nvsphngbm), and unclassified *Oxalobacteraceae* (UcOxlbtrc) in *Amubi* variety; while *Enterobacter* (Entrbctr)*, Caulobacter* (Clbctr), unclassified *Oxalobacteraceae* (UcOxlbtrc) were in *Poreiton*; and *Pleomorphomonas* (Plmrphmns)*, Bradyrhizobium* (Brdyrzbm)*, Novasphingobium* (Nvsphngbm) in Sempak. (Fig. 3). Five genera of phylum Proteobacteria *Shinella, Astacaccales* (Astccacls)*, Methylobium* (Mthlbm)*,* Unclassified *Comamonadaceae* (UcCmndc)*,* Unclassified *Sonobacteraceae* (UcSnbtrc)*,* were more abundant in young root than mature plants (Fig. 3). The abundance of the unclassified genus of the phylum Cyanobacteria and *Leptolyngbya* (Lptlnbya), unclassified *Pseudanabaenaceae* (UcPsdnbnc) remained constant in the young and mature shoot but decreased drastically in matured root compared to the young root (Fig. 3). However, the abundance of the genus *Planctomyces* (Plnctmyces)*, Gemmata* (Gmmta)*,* and unclassified Pirellulaceae (UcPrlc) of the phylum Planctomycetes in root and shoot decreases in the mature stage compared to the young stage (Fig. 3).

**Figure 4:**
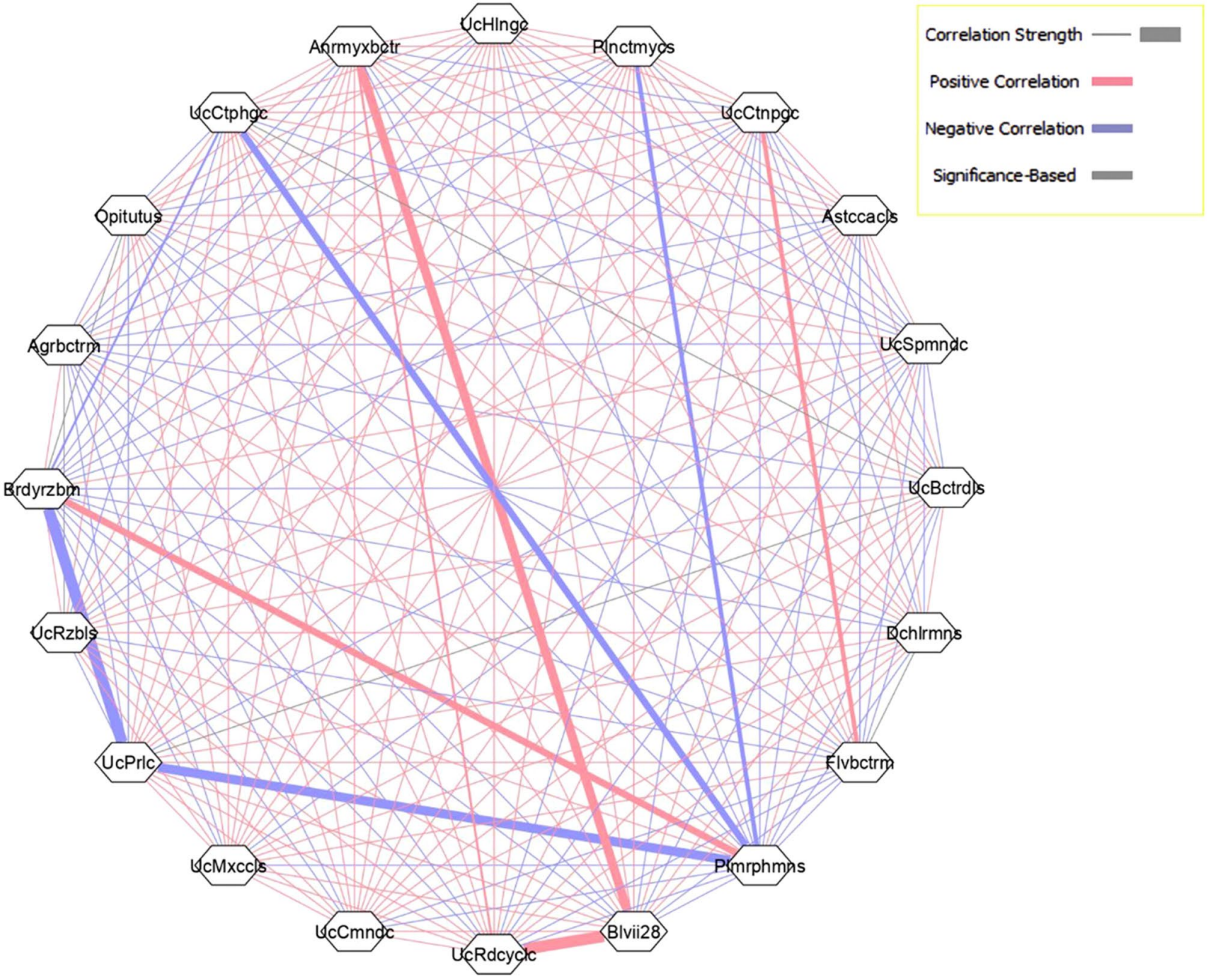
Network analysis revealing the co-occurrence patterns within the bacterial genera. Each of the nodes represents bacterial genera. The thickness of solid line (edge) between nodes denotes the strength of correlation (*P* < 0.05). Red lines indicate positive correlation while blue lines indicate negative correlation among the abundances of linked taxa. Network was visualized by Cystoscape V3.3.0.

The network analysis of bacterial genera confirmed that there are significant co-occurrence patterns among some genera are shown in (Fig. 4). The co-occurrence network was created with the strong and significant (*P* < 0.05) correlations, and the detailed information on correlation value is given in supplementary Table 3. As analysed, there was a significant Pearson rank correlation (*P* < 0.05) within the microbial diversity. Red lines indicate positive correlations while blue lines indicate negative correlations. The thickness of the lines indicates the strength of the correlation. A group of bacteria that showed a strong positive correlation were namely *Bradyrhizobium* (Brdyrzbm)- *Pleomorphomonas* (Plmrphmns); an *Anaeromyxobacter* (Anrmyxbctr)-Blvii28; Blvii28-unclassified *Rhodocyclaceae* (UcRdcyclc) while another group that showed a strong negative correlation with *Pleomorphomonas* (Plmrphmns) were Unclassified *Cytophagaceae* (UcCtphgc), unclassified *Pirullulaceae* (UcPrlc) and Planctomyces (Plnmyces).

Another group *Astacaccales* (Astccacls), *Anaeromyxobacter* (Anrmyxbctr), unclassified *Comamonadaceae* (UcCmndc), showed a general positive correlation among each other (*P* < 0.05). Overall, the genus that was abundant in the mature root of *Amubi* and *Poreiton* varieties of scented black rice plant showed a negative correlation with the genus that were abundant in the mature shoot of all three varieties. In the present study, it may be presumed that the microbial taxa could indicate some possible interaction among them and possessed significantly similar abundance patterns.

### Antioxidant property and its correlation with endosphere microbes through plant development

**Figure 5:**
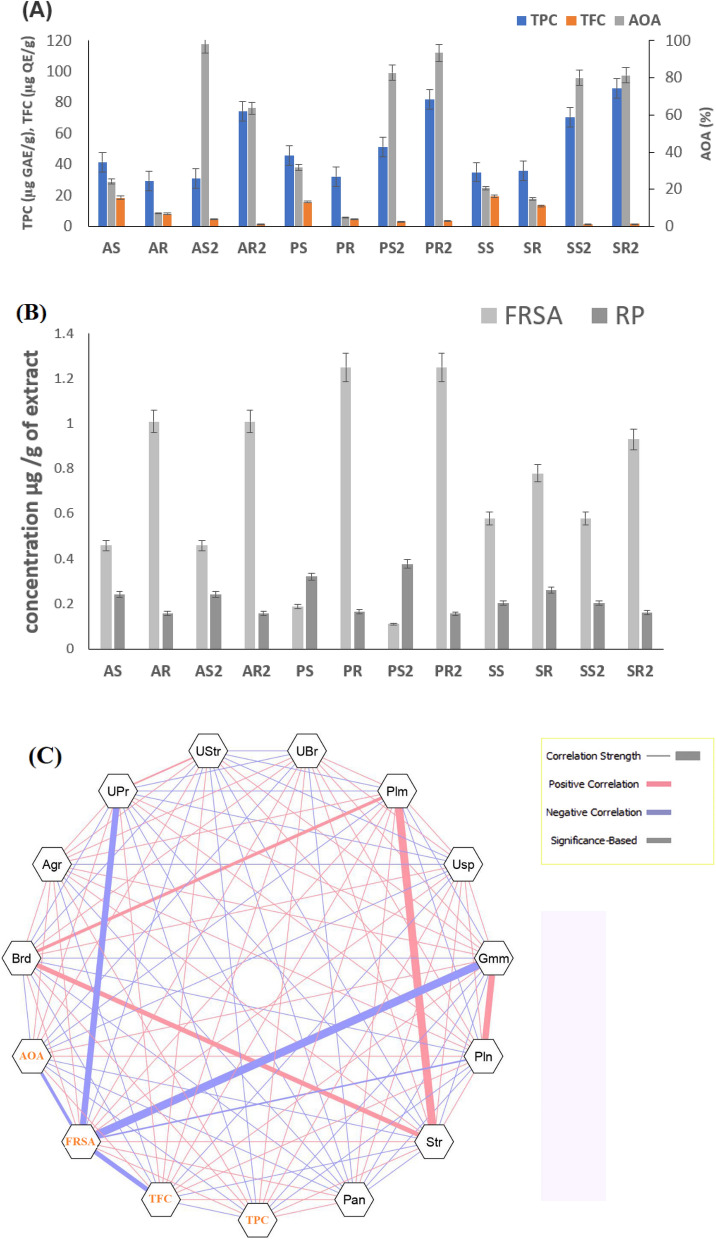
(**A**) Antioxidant assay TPC (µg GAE/g), TFC (µg QE/g) and AOA (%) in three variety of black scented rice. Graphs show mean ± SE. [AR-Young *Amubi* root, AR2-Mature *Amubi* root; AS-Young *Amubi* shoot, AS2-Mature *Amubi* shoot; PR-Young *Poreiton* root, PR2-Mature *Poreiton* root, PS-Young *Poreiton* shoot, PS2-Mature *Poreiton* shoot; SR-Young *Sempak* root, SR2-Mature *Sempak* root , SS-Young *Sempak* shoot, SS2-Mature *Sempak* shoot]. (**B**) Antioxidant activity (Free radical scavenging activity, FRSA) and (Reducing power, RP) of black rice. (**C**) Network analysis revealing the co-occurrence patterns between bacterial taxa and antioxidants (TPC,TFC,FRSA,AOA). Each of the nodes represents either bacterial genera or antioxidant activity. The thickness of solid line (edge) between nodes denotes the strength of correlation (*P* < 0.05). Red lines indicate positive correlation while blue lines indicate negative correlation among the abundances of linked taxa. Network was visualized by Cystoscape V3.3.0.

The TPC, TFC, and FRSA varied in root and shoot of different varieties of black rice plants at the young and mature stage of plant development (Fig. 5A). For three varieties *Amubi, Poreiton,* and *Sempak;* TPC ranged from 29.38 to 45.64 µg GAE/g (young) and 30.96–89.06 µg GAE/g (mature) (Fig. 5A). TFC in all the rice samples ranged from 4.72 to 19.34 µg QE/g and 1.28–4.55 µg QE/g in young and mature plants respectively. The AOA varied to a great extent, ranging from 4.89–31.89% in young and 63.80–98.02% in mature plants as measured by auto-oxidation of β-carotene and linoleic acid coupled reaction. It was observed that AOA was higher in mature plants, and among all, *Amubi* shoot had the highest AOA (Fig. 5A).

The IC50 values for DPPH free radical scavenging activity (FRSA) ranged from 0.10 to 1.25 (mg/ml), for the three varieties, among which, mature *Poreiton* shoot was observed to be higher (0.10 mg/ml) than those of other varieties. As IC50 is the concentration required to give half of the maximum inhibition, the smaller the IC50 means the better antioxidant activity (Fig. 5B). The reducing power RP was higher in mature *Poreiton* (0.321) and *Amubi* (0.242) shoot samples than in respective root samples (Fig. 5B). The statistical analysis of the samples has been attached in a supplementary Table 4.

To observe the correlation between the antioxidant compounds and the relative abundance of endophytic bacteria, correlation analysis was performed and visualized by network analysis (Fig. 5C). The correlation plot has been given in supplementary Fig. S5. To determine whether a specific class of antioxidant compound was influenced by a specific endophytic microbial community, the components of the endophytic community were correlated with TPC, TFC, FRSA, and AOA values.

The co-occurrences between bacterial taxa and antioxidant activity showed that abundance pattern of some genera is similar to that of the antioxidant activity, were further verified by correlation plot and network analysis. The correlation plot and values have been given in supplementary Fig. S5 and supplementary Table 5. To determine whether a specific class of antioxidant compound was influenced by a specific endophytic microbial community, the components of the endophytic community were correlated with TPC, TFC, FRSA, and AOA values. It was observed that the genus *Pleomorphomonas* (Plm) (corr value- 0.59) and *Streptomyces* (Str) (corr value-0.63) were positively correlated (*P* < 0.05) to TPC (Fig. 5C and supplementary Fig. S5) while TFC and AOA were found to be positively correlated (*P* < 0.05) with genus *Unclassified Pirellulaceae* (UPr), *Unclassified Stramenopiles* (UStr) and *Gemmata* (Gmm) (Fig. 5C and supplementary Fig. S5). While observing the free radical scavenging assay, the same genus *Pirellulaceae* (UPr), *Unclassified Stramenopiles* (UStr), and *Gemmata* (Gmm) were significantly negatively correlated to IC_50_ of DPPH this indicates these genera have strong IC_50_ value. Also, the genus *Pleomorphomonas* (Plm) was highly positively correlated to *Bradyrhizobium* (Brd) and *Streptomyces* (Str)*,* and *unclassified Bradyrhizobiaceae* (UBr) that were abundant in the mature plant of all varieties.

### The functional microbiome was influenced by plant development

**Figure 6:**
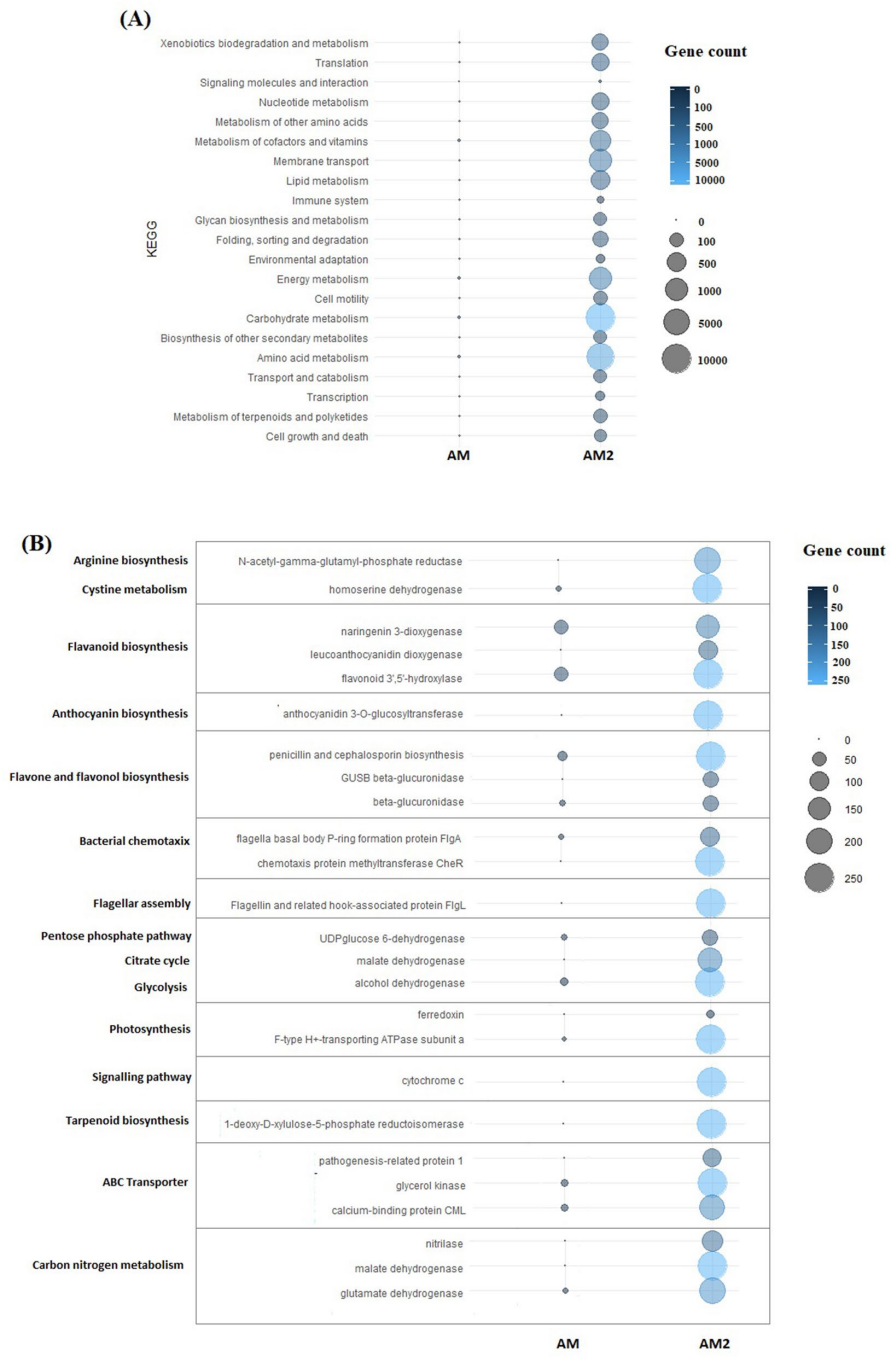
Bubble plots showing (**A**) KEGG functional categories of endophyte-based gene count in young and mature scented black rice plant. (**B**) Functional genes classified under hierarchical KEGG orthology present in the endomicrobiome at early (AM) versus late plant developmental time points (AM2). The size of the bubble signifies the gene count of each KEGG category function in young and mature stages. Colour chart indicator values are displayed.

From the KEGG analysis, it was observed that the majority of the bacterial functions and endophytic genes involved in secondary metabolite biosynthesis and another plant metabolism viz., nitrogen metabolism, the circadian rhythm of plants, terpenoid biosynthesis were more abundant at the mature stage (Fig. 6A). In this study, secondary metabolite biosynthesis that includes phenylpropanoid (flavonoid, flavonol, anthocyanin) biosynthesis has been focused on. The genes beta-glucosidase, naringenin -3-dioxygenase, and anthocyanidin 3-O-glucosyltransferase involved in flavonoid biosynthesis and anthocyanin biosynthesis, respectively were more abundant in the mature stage (Fig. 6B). Conversely, the gene nitrite reductase (NADPH) was more abundant during the plant young stage.

### Correlation of Antioxidant activity with the functional microbiome and enrichment of antioxidant (flavonoid, anthocyanin) producing genes through plant development

For evaluation of antioxidant activity that potentially mediates the functions carried out by the endophytic microbiome, gene-based evidence was carried out for a nearly complete phenylpropanoid (flavonoid and anthocyanin) synthesis pathway in the mature plant endophytic metagenome (supplementary Fig. S6). The genomic analysis for the particular gene assembly indicated that the genes that code for naringenin 3-dioxygenase (EC:1.14.11.9), naringenin chalcone synthase, and anthocyanidin3-O-glucosyltransferase [EC:2.4.1.115] can synthesize flavonoid and anthocyanin compound (supplementary Fig. S6). The BLAST analysis of the gene sequences suggested that some bacteria encode for the antioxidant activity, for example, the naringenin -3- dioxygenase gene codes for flavonoid biosynthesis was similar to that of *Streptomyces* sp. (NCBI accession no/gene id--LT629810.1/Ga0392509_019116_213_680). Similarly, the abundance of the beta-glucuronidase gene, which has a role in flavone and flavonol synthesis, was found to be higher in mature plants, and BLAST analysis revealed it to be similar to that of *Pantoea anannatis* (NCBI accession no/gene id--CP028033.1/Ga0392509_105731_7549_9309) (Supplementary Table 6). Moreover, the abundant gene contents related to the pathways of phenylpropanoid (Flavonoid and Anthocyanin) biosynthesis indicated that 4-Coumerate is converted to Dihydrokaempferol, and Leucodelphinidin to Anthocyanin, through the transformation of 4-Coumaroyl CoA to Naringenin chalcone and Isoliquiritigenin (supplementary Fig. S6A and S6B).

### Phenylpropanoid synthesis genes in endophytic isolates

**Figure 7:**
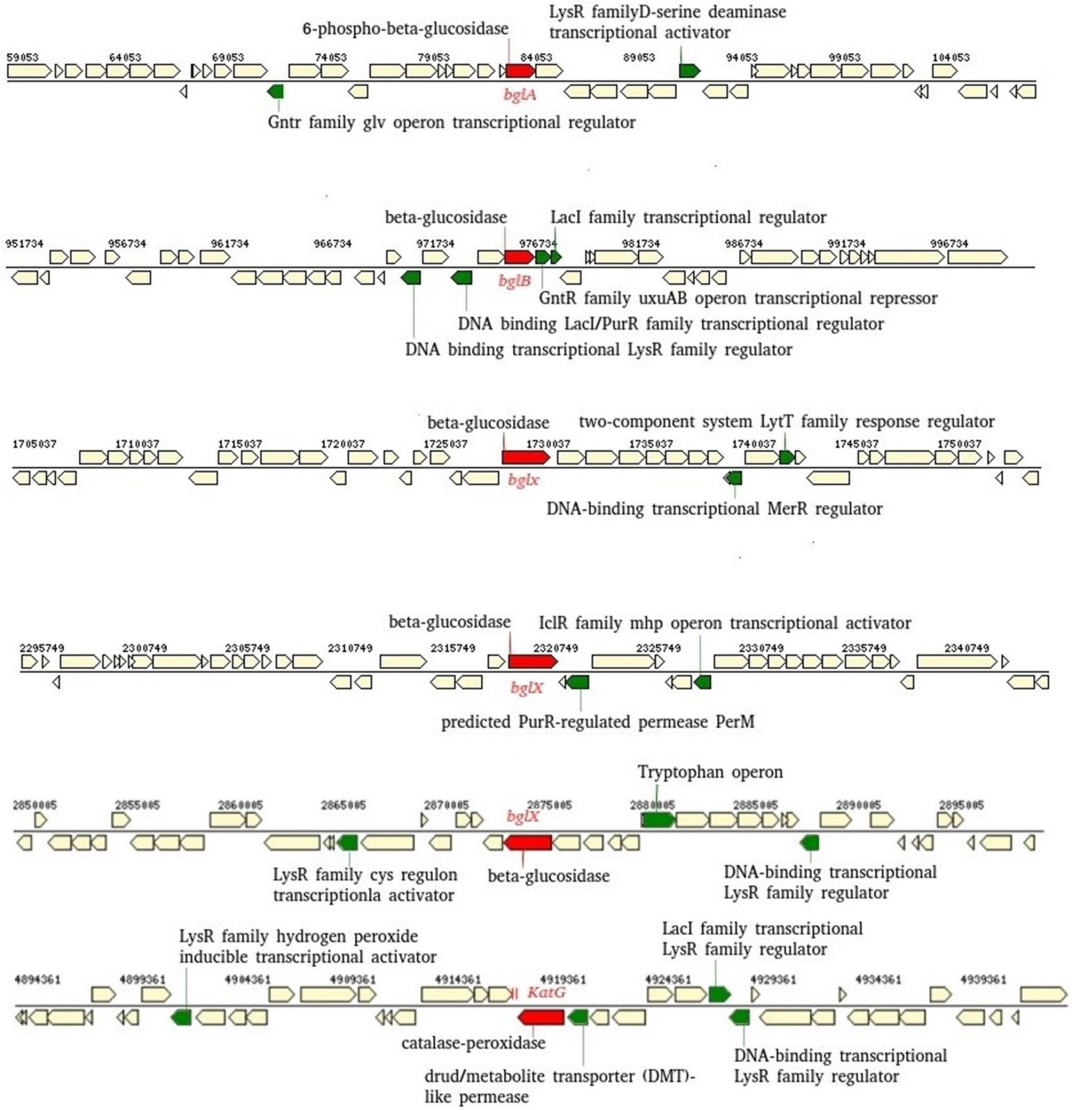
The phenylpropanoid synthesis (red) gene clusters in the assembled contigs in the genome of *Kluyvera sp.* Other regulatory genes are marked in green.

The gene ontology distribution for the genomes of *Kluyvera* sp. PO2S7, *Bacillus subtilis* AMR1 and *Enterobacter* sp. SES19 revealed that the genes involved in phenylpropanoid (antioxidant) synthesis and their regulatory elements were present in the genome. Several genes in the genome of *Kluyvera* sp. PO2S7 (CP050321), *Bacillus subtilis* AMR1(CP050319) and *Enterobacter* sp. SES19 (CP050320) namely 6-phospho-beta-glucosidase 83357..84745 (*bglA*), beta-glucosidase 976019..977446 (*bglB*), beta-glucosidase 1728887..1731184 (*bglX*), beta-glucosidase 2319561..2321933 (*bglX*), beta-glucosidase 2873828..2876179 (*bglX*), catalase-peroxidase 4918272..4920446 (*katG*) were located in distinct genomic regions. The elements responsible for phenylpropanoid biosynthesis were organized as an operon separately with transcription regulator as given for the whole genome sequence of *Kluyvera* sp. (Fig. 7). The genome also contained *LysR* as a transcriptional activator gene, located adjacent to *bglA* in the same orientation, and *GntR,* operon transcriptional regulator of bglA located in the opposite orientation. Whereas *GntR* family uvxAB operon transcriptional repressor and *LacI* transcriptional regulator were present adjacent to the beta glucosidase (*bglB*) gene. Besides, in the case of the beta glucosidase gene (*bglX*), the MerR operon was regulated by the *LytT* regulator. The gene cluster arrangement is given (Fig. 7).

## Discussion

The endophytic microbial community at the root and shoot of the young stage was very different from the mature-stage black rice plant (Fig. 1). A similar study by van Overbeek^16^ confirmed that the significantly different endophytic bacterial communities of potato plants at different stages of plant development, affected the dynamic structure of the endophytic bacterial community in potato plants. Hence, this indicate that scented black rice plant growth stages affected the endophytic bacterial communities. A comprehensive study of the assembled endosphere microbial communities of black rice plant maturation resulted in an established core microbiome (Fig. 2A), which was comprised of the phylum Proteobacteria (Fig. 2B i), Cyanobacteria (Fig. 2B ii), and Planctomycetes (Fig. 2B iii), but their abundance changed significantly at the mature stage, in all three varieties (*Amubi, Poreiton*, and *Sempak*). Likewise, Ferrando et al^17^ observed some common endophytes in two consecutive rice crop seasons which proposed a strong correlation between these bacteria and the plant. Also, it had been suggested that the colonization efficiency of endophytic bacteria changes with the growth of the host plant, and the latter selects a set of microbes according to its requirements^18^, which may be correct for scented black rice plants as the same was observed for all the three varieties.

Despite the presence of several common phyla in the black rice plant, the relative abundance of Proteobacteria was found to be highest in roots of mature plants, and its abundance varied greatly between shoot to root (Fig. 2B, i). Also, the phylum Proteobacteria has been reported to be the endophytic dominant phylum in crops, such as wheat and rice^19,20^. The predominance of Proteobacteria in various plant samples could be due to the utilization of organic acids^21^.

Some of the genera within Proteobacteria i.e., *Pleomorphomonas, Bradyrhizobium, Novasphingobium* (mature *Amubi* and *sempak* root); *Caulobacter* (mature *Poreiton* root) were more abundant in the mature stage of the black rice plant (Fig. 3). In the study of Okunishi et al^22^ the genera *Burkholderia, Enterobacter,* and *Pantoea* were reported to be abundant in mature rice, which was different from our observations with the scented black rice plant. Some of the genera which were detected in the core microbiome of the scented black rice plant are well-established plant growth-promoting rhizobacteria (PGPR), that are known to promote plant secondary metabolites accumulation and consequently, antioxidant capacity^23,24^ such as *Bradyrhizobium, Streptomyces,* Rhizobium. These genera had higher abundance in the root of the mature stage in all the three varieties- *Amubi, Poreiton,* and *Sempak*. The abundance of *Agrobacterium* sp. that help in nitrogen fixation was found to be higher in the mature stage in black rice *Amubi* variety, which is similar to a previous report from hemp plant^25^. The variation in the abundance of some endophytic genera with plant development might be due to the ability of the plants to recruit a particular community of bacterial endophytes by the composition and concentration of sugars and amino acids derived from the plant at each developmental stage^26^. Therefore, endophytic bacteria may help in plant growth promotion and have the ability to colonize and grow inside the black rice tissues and be highly adapted to the plant niche.

Mano et al^27^ reported a similar result with the present study where a higher abundance of endophytic bacteria was observed in roots compared to shoots. Comparable observations were also found while studying the various cultivated crops, for instance, potato, maize, and rice^19^. Robinson et al^28^ had ascribed roots as desirable favorable places for endophyte colonization as roots are the reservoir for photosynthetic carbon, and protected from excesses of temperature, solar radiation, and moisture variations.

The members of Cyanobacteria are known to colonize plant roots^29^ and promote plant growth and providing C and N nutrition to the host^30^. Chaparro et al^18^ observed a higher abundance of Cyanobacteria in the mature stage of the *Arabidopsis* plant as compared to the seedling stage. Cyanobacteria are a diverse group of photosynthetic and nitrogen-fixing bacteria and remain abundant in the shoot that performs photosynthesis and therefore, members of Cyanobacteria, recruit to shoot for photosynthesis and providing C and N to the host is considered as an adaptation for the favorable environment^31^.

On the other hand, the genera of Phylum Planctomycetes *(Planctomyces, Gemmata, Unclassified Pirulullaceae, Unclassified WD2101*) significantly decreased in mature stage plants in comparison to young stage plants (Fig. 3) yet the role of *Planctomycetes* in the endosphere has not been elucidated. Ferrando^17^ suggested the reason for the decrease in specific endophytes in rice was due to translocation of carbohydrates from shoot to grains and decrease of nutrients in shoots that become less accessible for some bacteria during the mature stage.

From the co-occurrence analysis (Figs. 3, 4), the presence of particular genera and their negative correlation with the other genera indicated probable selection of particular bacterial genera by the endophytic community during plant development^18^. Agler et al^32^ had proposed that some dominant microbes in the community act as hubs and have a strong negative correlation with networks of many microbial communities. Such interaction was sensed in the endophytic microbiome of black rice.

Black scented rice is especially rich in anthocyanin pigments, phytochemicals, protein, and vitamins^10^. In cereal grain, one of the major and most complex groups of phytochemicals is the phenolic compounds^33^. In most of the previous research, the TPC has been estimated in the grains of black rice which were found to be almost 50 times higher^34^ than the values recorded in the shoot of three varieties, though information on the phenolic content of shoots of black rice is not available. In the present study, it was observed that the TPC of the mature root of black rice plants was higher in comparison to young plants (Fig. 5A). A similar observation has been reported incarnation, where levels of phenolics and flavonoids were higher in the roots than in the stems^35^. As described by Fico et al^36^, the variation in antioxidant compounds was due to dependence of regulation of the biosynthesis of these compounds on the plant part and its adaptational needs; which might be true for scented black rice plant samples.

In all three varieties, the higher TPC content was found to be correlated with the abundance of some genera for example *Pleomorphomonas* (Plm) and *Streptomyces* (Str). Besides, from the correlation study, some genera such as *Gemmata* (Gmm), *Unclassified Pirellulaceae* (UPr), were highly negatively correlated to FRSA. A negative correlation to IC_50_ of DPPH indicated that these genera have a strong IC_50_ value. Rahman et al^37^ observed an increase in total flavonoid, phenolics content, total antioxidants in the strawberry plant after application of *Bacillus* and probiotic strains. Nakaew et al^13^ observed a link between the richness of bioactive phytochemicals; anthocyanin, phytate, and antioxidants with bio functions of endophytic actinobacteria, and reported that phytochemicals and endophytic community structure were closely related in rice plant in various stages, which is similar to our observation with black rice varieties.

Most of the endophytic genes, associated with various plant functions were found to be significantly abundant at the mature stage (Fig. 6A, B). The plant-associated microbiomes had been found to get enriched with a higher abundance of microbial genes in the mature stage than in the young stage^18^. In a previous study by Chaparro et al^18^, it was found that the abundance of a functional gene may be altered through plant maturation, even when the abundance of any bacterium carrying out that function did not change much. However, in the present study, the relative abundance of some bacterial genera namely *Streptomyces* increased as the plants matured, and the abundance of naringenin-3-dioxygenase genes that code for phenylpropanoid (flavonoid and anthocyanin) also increased. Moreover, endophytic bacteria (*Streptomyces*) were typical endophytes of rice that were well known as a source of bioactive metabolites in rice plants. Also, the total antioxidant activity was recorded to be higher in mature black rice plants (Fig. 5a). Although, it's early to state that the abundance of naringenin 3-dioxygenase phenylpropanoid synthesis genes has a definite role, yet both these observations seem interesting when put together. Rahman et al^37^ also described the secretion of bioactive compounds by endophytes that dependent on the secretion of plant secondary metabolite and enhanced antioxidant production in strawberry fruits. In the study of Chamam et al^38^ *Azospirillum* sp. was found to modify the phenolic compounds in rice and reported that symbiosis induced synthesis of phenolics and have an effect on the secondary metabolism in plants. Therefore, the genes present in the endophytic microbial community of black rice plants indicate that they might induce/have a role in the enhancement of the antioxidant activity in scented black rice plants.

To date, almost ten thousand flavonoids have been identified in plants, and their synthesis appears to be ubiquitous^39^. Analysis of the functional gene in the endophytic microbiome of black rice indicated an approximately complete flavonoid and anthocyanin (phenylpropanoid) biosynthesis pathway present in the endophytic bacterial community (supplementary Fig. S6). The pathways for the flavonoid and anthocyanin (phenylpropanoid) biosynthesis were reconstructed through KEGG pathway analysis^29,40^ where the genes involved in the synthesis process were found to be the bacterial origin, even though the flavonoid biosynthesis pathways are common in plant^41^ whereas in bacteria these pathways are known to be less common.

Comprehensive genome analysis of three potential endophytic isolates (*Kluyvera* sp. PO2S7, *Bacillus subtilis* AMR1 and *Enterobacter* sp. SES19 also confirmed the presence of phenylpropanoid synthesis genes (*bglA*,*bglB*, *bglX*, *katG*) in their genomes. According to Safdarian et al^42^ P450s genes (*CYP98A1*, *CYP734A5*, *CYP72A15*, and *CYP710A1*) served as the signals for growth and development for protecting plants from different biotic and abiotic stresses were present in the phenylpropanoid biosynthetic pathway. Safdarian et al^42^ had suggested that inoculation of potential endophyte enhances the antioxidant enzyme activity, which also has a significant role in bacteria-mediated salt tolerance of host plants. So, from the description of genomic organization and function analysis, it may be assumed that beneficial endophytic bacteria help the host plant in the synthesis of phenylpropanoid. This requires more experiments for validation.

Ali et al^43^ confirmed the increased transcriptional profile of phenylpropanoid pathway genes and increased contents of flavonoids in Arabidopsis after application of microbial products. Zhang et al^34^ and Taghinasab et al^24^ described that for synthesizing secondary metabolites including antioxidants, endophytes and hosts acquire similar pathways due to gene transfer and might be due to the existence of the same niche, and through continuing co-occurrence and direct interaction, they have exchanged genetic material. Moreover, metabolic interactions between endophytes and their hosts may induce the synthesis of active secondary metabolites^44^ and give endophytes a competitive benefit in the endosphere^45^.

Khare et al^46^ reported that both the plant and their endophytes could produce an array of common secondary metabolites from similar precursors. Therefore, it was assumed that the scented black rice endophytes use a common phenylpropanoid (flavonoid and anthocyanin) synthesis pathway, similar or different to plant, and the high antioxidant activities of black rice are a function, which is mutually shared with the endophytic microbiome. Specific enrichment of the antioxidant-producing genes and their function in the mature plant endophytes suggested that some of the endophytic bacteria might be an important provider of these genes for the antioxidant activity of the host plant. However, this is a primary observation-based on the presence of genetic elements, and the role in an antioxidant activity needs further research. Therefore, the plant–microbe interaction may be exploited to increase the production of phytochemicals in scented black rice, and hence need to be studied further.

## Conclusion

This study provides insights on the endophytic microbiome of black scented rice, which has not been characterized previously. The conclusions of the present study could be stated as- (a) Black rice plant sustains a core endophytic microbiome which varies between young and mature stage of plant development, as assessed for the three different varieties. (b) The loss of overlap in community structure during the growth of the plant (young to mature) within the root and shoot tissues of black rice plants indicated dynamic nature of the community. (c) The presence of antioxidant (phenylpropanoid) synthesis genes in both endophytic microbiome and genome of potential culturable endophyte suggest that there is a possibility that the endophytes contribute towards the antioxidant activity. In general, these concepts suggest that plants and the endophytic microbiome perform similar functions through a common biosynthetic pathway by gene transfer. More extensive studies are needed to decisively determine the interactive functions that occur in the black rice plants and their endophytic microbiome.

## Materials and methods

### Plant sample collection and DNA extraction for metagenomic analysis

Three replicate plants of the three black rice varieties (*Amubi, Poreiton,* and *Sempak*) at young (1 month after planting) and mature (6 months after planting) stages were collected from Central Agricultural University (CAU), Manipur, India located in North-Eastern of the Indian subcontinent latitude ranging from 23°83′N–25°68′N and longitude 93°03′E–94°78′E, annual rainfall varies from 1467.5 to 2593 mm where plants were grown. The soil properties were checked following the protocol of Nayak^47^ "The soil was acidic (pH varied from 4.5 to 5.4), derived from sedimentary rocks, low cation exchange capacity (CEC) and much less content of sand but had moderate to high amounts of silt and clay fractions. The minerals present in the clay fraction of the soils were similar to those of the silt consisting of mica (1.0 nm) and kaolin (0.72 nm) minerals. The macronutrients present in soil were NH_4_^+^, H_2_PO_4_^−^, K ^+^, and SO_4_^--^ and micronutrients, that is, Zn^+^, Cu^+^, Mn^++^, Fe^++^, and B^+++^ which is in accordance with the previous report^47^.

Ten samplings (three replicates) of each plant variety were randomly taken in triplicate by using a clean spade to remove intact roots from the soil. The samples were collected in the sterilized package and immediately transported, on ice, to the laboratory for microbiological analyses.

### Surface sterilization and DNA extraction from the black rice plant samples

Triplicate portions of separated stems and roots of black rice plant were subjected to surface sterilization by immersing in 70% (v/v) ethanol for 3 min, followed by 2.5% (v/v) sodium hypochlorite (NaOCl) for 5 min by following the protocol of Barra et al^48^. Roots and shoots were thoroughly rinsed with sterile distilled water. Triplicate portions of roots and leaves were aseptically cut, soaked, and homogenized with a mortar and pestle, and stored at − 80 °C until DNA extraction. The homogenized tissues were used for DNA extraction E.Z.N.A. HP Plant DNA Kit (Omega) according to the manufacture’s instruction^24^. The quality of DNA extracts was checked by measuring absorbance at 260 nm and 280 nm by using a microplate spectrophotometer (Multiskan GO, Thermo Fisher Scientific, Inc., MA, USA).

To attain information about endophytic community composition of root and shoot of three varieties of black rice (*Amubi, Poreiton,* and *Sempak*), and also functional roles of the endophytic community, we performed amplicon, as well as shotgun sequencing respectively.

### Amplicon sequencing for taxonomic assignment of metagenomic sequences

Nextera XT Index Kit (Illumina inc.) was used for the preparation of the amplicon library as per the 16S metagenomic sequencing (Illumina inc.). The 16S rDNA gene targeting the V3-V4 region precise for bacteria was amplified using the specific primers (16S rRNA F-GCCTACGGGNGGCWGCAG) and (16S rRNA R- ACTACHVGGGTATCTAATCC). PCR reactions of all the samples were carried out in triplicate. The libraries were sequenced on MiSeq using a 2 × 300 bp paired-end manner. As per the standard Illumina protocol, the amplification of the amplicons with the Illumina adaptors was completed by using i5 and i7 primers for cluster generation (P5 and P7). Purification of the amplicon library was done by 1× AMpureXP beads and quantified using a Qubit fluorometer. In the 4200 Tape Station system (Agilent Technologies), the amplified libraries were analyzed by D1000 Screen tape according to manufacture directives. After that, at an appropriate concentration (10–20 pM), libraries were loaded onto MiSeq for cluster generation and paired-end sequencing. On MiSeq, the template fragments were sequenced in both the forward and reverse directions in the paired-End sequencing. In the binding of samples to complementary adapter oligos, the kit reagents were used on the paired-end flow cell.

### Data processing, bioinformatics, and statistical analysis

On Illumina MiSeq platforms, amplicon sequencing was performed at Eurofins Genomics India Pvt. Ltd. From the sequencing process, the raw data resulted was transported into FASTA files for each sample, together with sequencing quality files. By using the bioinformatics software, the Quantitative Insights Into Microbial Ecology (QIIME), files were accessed. Quantitative Insights into Microbial Ecology QIIME2 (version 2019.7) pipeline was used to analyze the paired-end sequences and to produce the taxonomic abundance of microbial community^49^. Paired-end sequences were merged to get the full length of the fragments using QIIME. The resulting paired sequences were demultiplexed based on the unique barcode, and potential PCR chimeras’ sequences were removed. Trimmomatic v 0.35 was used to eliminate adapter sequences from the sequence reads. Ambiguous reads and low-quality sequences (read with more than 10% quality threshold (QV) < 20 Phred score) were screened for contamination with rice plant DNA using megablast against the *O. sativa* genome. To remove the effect of non-microbiota (e.g., chloroplast and mitochondria), the sequences were further filtered by QIIME. The sequencing reads obtained in each sample were given in Supplementary Table 1. By using the Uclust algorithm at 97% sequence similarity, sequences were clustered into operational taxonomic units (OTUs)^50^. Finally, the RDP (Ribosomal Database Project) classifier was used to assign the representative sequence to the microbial taxa based on a threshold of 97% sequence similarity^51^. For analyzing data, open-reference OTU picking was used. By aligning to a reference database, or the read that does not match an identified sequence is referred for de novo OTU picking. through OTU picking OTU information created was used for estimation of diversity within and between samples. Principal Coordinate Analysis (PCoA) was carried out to measure how similar or dissimilar the samples are. Each point is represented by a sample and the distance between the points represented the similarity of those samples.

The METAGENassist was used to perform multivariate data analysis of the OTUs^52^, and subsequently, normalization based on interquartile range (IQR) and log2-transformation^18^. Principal component analysis (PCA) and significant features were calculated for all samples using METAGENassist^52^. PCA measures variances in the dispersal of taxonomic classifications between samples, up to a fixed taxonomic level. The Vegan package^53^ for R was used for community dissimilarity calculations (Bray–Curtis index) and principal coordinate analysis (PCoA). The heatmap of the 50 most abundant OTUs at the genus level in each sample was constructed by using METAGENassist^52^. The heat map represents the relative abundance of the separate bacterial genus within each sample. The data is presented on a web where each row represents a genus and each column represents a sample. The intensity and color of the boxes are used to signify relative values (Z-score values) for the bacterial genera. The mean value is represented by the Zero on the color scale. The value + 3 represents two standard deviations above the mean and the value − 3 represents two standard deviations below the mean. The red color signifies abundant genera and the blue represents less abundant genera.

Diversity indices were determined by PAST software. The correlation analysis within bacterial genera; between antioxidant activity and bacterial genera was performed by using R Vegan package^53^.

### Functional analysis (Shotgun sequencing)

To understand the functional role of the endophytic community at young and mature stage plants, a shotgun library was prepared by using the TruSeq Nano DNA Library Prep Kit. Illumina library was loaded onto NextSeq 500 for cluster generation and sequencing. Paired-End sequencing of the template fragments to be sequenced was performed by using Illumina NextSeq 500. Low-quality sequences were removed, screened for contamination with rice plant DNA using megablast against the *O. sativa* genome. The filtered metagenomic reads were used for taxonomical assignment by the Kaiju web server for the identification of microbial species in the plant samples using NCBI BLAST taxonomy data sets as a reference database. The output of the community analysis through shotgun sequencing, was generated as Krona graphs. The total functional high-quality reads 9,559,503 (young) and 17,545,129 (mature) were obtained for further assembling. The filtered high-quality reads were assembled into scaffolds using CLC Genomics Workbench version 9.5.2^9^. Prodigal-2.6.3 with default limitations was used to envisage the genes from assembled scaffolds. Cognizer was used to carry out the functional analysis of the genes from the sample, enabled to concurrently run COG, KEGG, Pfam, GO and SEED subsystem annotations to individual sequences creating metagenomic datasets. The use of a novel 'directed search' step in COGNIZER significantly reduces the overall compute requirements typically associated with functional analysis. The final metagenomic assembly was uploaded into MG-RAST pipeline version 3.3^9^ and IMG^54^ separately, or for gene prediction and annotation. BLAST analysis of the particular gene sequences responsible for desired functions was performed to assume genes specific to the bacteria. Antioxidants like polyphenol, flavonoid, and anthocyanin were annotated based on secondary metabolite biosynthesis distribution in KEGG databases^40,51^. The annotations for all predicted antioxidants were inspected manually, counted, and named. The Vegan package for R was used for the bubble diagram that signifies variation of particular genes in young and mature stages of plant growth. The R scripts for correlation study and bubble diagram have been attached in the supplementary R script. R1, R2, and R3.

### Total polyphenol content (TPC) and total flavonoid content (TFC)

Total polyphenol content (TPC) and total flavonoid content (TFC) were determined according to the methods of ^55^ respectively. Extracts prepared from 200 mg of stems and roots using 1 mL of 80% (v/v) methanol were used for further analysis. The methanolic mixtures were then sonicated for 15 min (42 Hz and 100 W) and centrifuged (12,000 g, 15 min). The supernatants were stored in the dark at − 70 °C for subsequent analysis. For each variety, all analyses were performed in triplicate.

To estimate TPC, aliquots (1.0 mL) of properly diluted extracts were mixed with 1 mL of 1 N Folin-Ciocalteu reagent and the reaction was neutralized with 2 ml of saturated sodium carbonate (20 g/100 ml). The absorbance of the subsequent blue color was noted at 760 nm using a spectrophotometer after incubation for 2 h at 23 °C. By using a gallic acid standard curve (0–100 µg/mL) as the standard, TPC was determined and expressed as µg of gallic acid equivalents (GAE; Sigma-Aldrich) /g of the formulation.

To estimate TFC, Aliquots (1 mL) of properly diluted extracts were pipetted into polypropylene conical tubes comprising 2 mL of double-distilled H_2_O and mixed with 0.15 mL of 5% NaNO_2_. 0.15 mL of 2% AlCl_3_·6H_2_O solution was mixed after 5 min and allowed to stand for another 5 min, then 1 mL of 1 M NaOH was added. The reaction solution was mixed and kept for 15 min; absorbance was determined at 415 nm. Total flavonoid content was calculated by Quercetin (0–100 µg/mL) standard curve and expressed as mg of quercetin equivalent µg (QE)/ g of formulation.

### Free radical scavenging activity (FRSA)

By using 1,1-diphenyl-2-picrylhydrazyl stable radical, the DPPH free radical scavenging activity was assayed as described^56^. 100 μmol/L of DPPH radical solution was prepared in methanol. To the 3 ml DPPH solution, properly diluted crude extracts of 2–10 mg/ml (0.1 mL) were added. The absorbance was measured after incubating for 30 min in the dark with the help of a spectrophotometer at 517 nm.

The absorbance of the control and samples was measured, and the DPPH scavenging activity was determined (in percentage), which was calculated according to the following formula:

$${\text{Scavenging}}\;{\text{effect}}\left( \% \right) = \left[ {\left( {{\text{AC}}{-}{\text{AS}}} \right)/{\text{AC}}} \right] \times 100$$

where Ac: absorbance of the control, As: absorbance of the sample (extract). The data are presented as the mean of triplicate and the concentration required for a 50% (EC50) reduction of DPPH radical as determined with the help of a standard graph. The reduction of the DPPH radical was measured continuously until constant values were obtained and it was expressed in terms of inhibitory concentration IC_50_ (mg/mL).

### Reducing power (RP) assay

RP of the extracts was determined by using the modified ferric reducing-antioxidant power assay^57^. 1 ml of the extract was mixed with 2.5 ml of phosphate buffer (0.1 M, pH = 6.6) and 2.5 ml of 1% potassium ferricyanide, and incubated at 50 °C for 20 min. In this mixture, about 2.5 ml of trichloroacetic acid (TCA) (10%) was added and the solution was centrifuged for 10 min (3000 rpm). Finally, 2.5 ml of the supernatant solution was mixed with 400 μl of distilled water and 0.5 ml FeCl_3_ (0.1%), and the absorbance of the final-colored solution was measured at 700 nm.

### Antioxidant activities (AOA)

According to Singh et al^55^, the 3 ml of the reaction mixture containing 2 mg of β carotene dissolved in 20 mL chloroform was added to 0.1 mL of extract. 40 mg of linoleic acid was added to 400 mg of tween 40 emulsions. To the 80 µL of formulation solution (1 mg mL^−1^), the 3 mL aliquot of the β-carotene and linoleic acid were mixed and incubated at 50 °C. The reaction mixture was kept at room temperature for 6 min and the absorbance was recorded at 734 nm, with reference to control, AOA was expressed as percent inhibition relative to control.

### Correlation of microbiome to antioxidant activity/ gene prediction and functional characterization and validation

For validation, culturable endophytes were isolated according to the method of Taghavi et al^58^. From the culturable isolates, the three most potential culturable endophyte *Kluyvera* sp. PO2S7, *Bacillus subtilis* AMR1and *Enterobacter* sp. SES19 was selected based on PGP attributes, and pot trial experiment (data not given) and whole-genome analysis has been carried out according to Safdarian et al^42^. To evaluate the function of antioxidant (phenylpropanoid) synthesis genes, the annotated coding sequences were assigned to the IMG server. By using the NucleoSpin DNA Extraction Kit for DNA (NucleoSpin, Germany), genomic DNA from the *Kluyvera* sp. PO2S7, *Bacillus subtilis* AMR1and *Enterobacter* sp. SES19 strain was extracted from exponential growth cultures (1 mL, A600 = 0.5). In Nano-drop 2000 (Thermo Scientific Inc, USA), the quality and quantity of genomic DNA were checked by determining the A260/280 ratio. DNA concentration was checked by Qubit 3.0 Fluorometer (Thermo Scientific Inc, USA) for library preparation. Genome sequencing was performed at Eurofins Genomics India Pvt. Ltd. with the paired-end sequencing libraries prepared using TruSeq Nano DNA Library Prep Kit for Illumina (NextSeq-500 libraries). The library fragment size disseminations were subjected to end-repair after that adapter ligation to the fragments was done. By using AMPure XP beads, the ligated products were size-selected and used in PCR amplification using the index primer. In Tape Station 4200 (Agilent Technologies, USA), the PCR amplified libraries were analyzed using High sensitivity D1000 Screen Tape assay kit as per manufacturer instructions. High-quality paired-end short reads of *Kluyvera* sp. PO2S7, *Bacillus subtilis* AMR1and *Enterobacter* sp. SES19 attained from Illumina NextSeq-500 were amassed into scaffolds by using SPAdes (Version: 3.7.1) with default parameters^59^.

### Network analysis

The network analysis was performed using the Hmisc packages of R 3.4.2. In brief, the pairwise Pearson's correlation coefficients (r) between bacterial taxa; bacterial taxa, and antioxidant activities were calculated based on the relative abundance of bacterial genera and antioxidant values. R and *P* values were generated using the R package hmisc and were adjusted with a multiple testing correction using the Benjamini–Hochberg method to reduce the chances of obtaining false-positive results. Cytoscape 3.3.0 software (http://cytoscape.org/) was applied to visualize the network graph.

### Statistical analyses

The mean and standard error for each set of data for relative abundance and antioxidant activities were calculated. The diversity Dominance, Evenness, Shannon, Simpson index, was calculated by using PAST software. To check significant differences among the endophytic community, ANOVA and PERMANOVA was performed by PAST software. Correlation analysis was performed to identify the influence of endophytic microbial community on antioxidant compounds. R statistical software and Cytoscape were used to visualize correlation and bubble diagram.

### Nucleotide accession number

Sequence data have been deposited in NCBI Sequence Read Archive (SRA) under the accession number SRR8047931, SRR8047932, SRR8047933, SRR8047934, SRR8047930, SRR8047929 (under the Bioproject No. PRJNA495908); SRR7619128 (under the Bioproject No. PRJNA483371); SRR8051584, SRR8051586, SRR8051585, SRR8051582, SRR8051581, SRR8051583 (under the Bioproject accession No. PRJNA482103) and SRR8089794 (under the Bioproject accession No. PRJNA496258); CP050321 (PRJNA604081).

## Supplementary Information

Supplementary Information.
