# Supplementary material for: Host specific endophytic microbiome diversity and associated functions in three varieties of scented black rice are dependent on growth stage
Source: Sci Rep. 2021 Jun 10;11:12259. doi: 10.1038/s41598-021-91452-4 (PMC8192550; doi:10.1038/s41598-021-91452-4)
Supplement: Supplementary file 1 — Supplementary Information. [file 41598_2021_91452_MOESM1_ESM.docx]

**Title:Host specific endophytic microbiome diversity and associated functions in three varieties of scented black rice are dependent on growth stage.**

**Authors:**

K. Malabika Singha

Department of Microbiology,

Assam University, Silchar- 788011

malabikasingha9@gmail.com

Brahmanand Singh

Department of Pharmacognosy and Ethnopharmacology.

CSIR-National Botanical Research Institute

Lucknow,Uttar Pradesh, India-226001

bn.singh@nbri.res.in

Piyush Pandey

Department of Microbiology,

Assam University, Silchar-788011

ppmicroaus@gmail.com

**Corresponding Author: Prof. Piyush Pandey**

**Email Address:** [**piyushddn@gmail.com**](mailto:piyushddn@gmail.com)


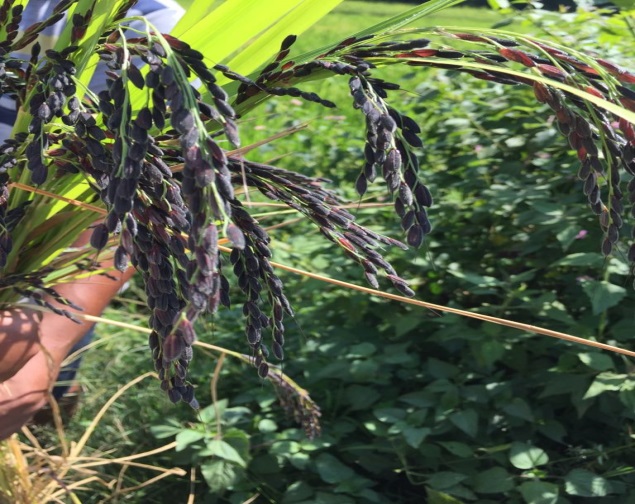

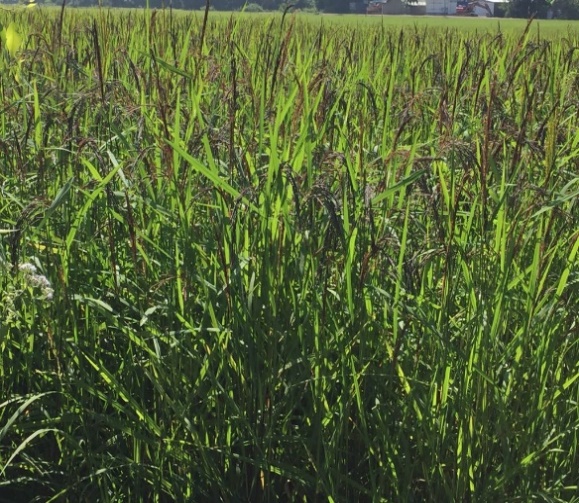


**Supplementary Fig. S1** Black rice plant growing in the field of Central Agricultural University, Manipur.

**Supplementary Table 1**---Data statistics of samples

| Sl no. | Sample | #Reads | Total bases |
| --- | --- | --- | --- |
| 1 | AS (young *Amubi* shoot) | 364,514 | 188,801,528 |
| 2 | AR (young *Amubi* root) | 357,297 | 185,324,882 |
| 3 | AS2 (mature *Amubi* shoot) | 534,887 | 261,458,995 |
| 4 | AR2 (mature *Amubi* root) | 397,964 | 199,120,289 |
| 5 | PS (young *Poreiton* shoot) | 392,803 | 203,007,502 |
| 6 | PR (young *Poreiton* root) | 423,106 | 220,586,543 |
| 7 | PS2 (mature *Poreiton* shoot) | 212,525 | 105,468,578 |
| 8 | PR2 (mature *Poreiton* root) | 171,400 | 101,213,329 |
| 9 | SS (young *Sempak* shoot) | 335,511 | 172,728,853 |
| 10 | SR (young *Sempak* root) | 458,600 | 240,696,210 |
| 11 | SS2 (mature *Sempak* shoot) | 444,998 | 215,890,165 |
| 12 | SR2 (mature *Sempak* root) | 309,787 | 154,715,387 |

**Supplementary Table 2**-Diversity indices of the samples

|  | **AS** | **PS** | **SS** | **AR** | **PR** | **SR** | **AS2** | **PS2** | **SS2** | **SR2** | **AR2** | **PR2** |
| --- | --- | --- | --- | --- | --- | --- | --- | --- | --- | --- | --- | --- |
| **Dominance** | 0.4526 | 0.1057 | 0.3105 | 0.06078 | 0.1856 | 0.1318 | 0.4594 | 0.6467 | 0.7057 | 0.101 | 0.0773 | 0.2421 |
| **Simpson** | 0.7695 | 0.8943 | 0.7895 | 0.9392 | 0.8144 | 0.7532 | 0.8567 | 0.8281 | 0.9243 | 0.899 | 0.9227 | 0.7579 |
| **Shannon** | 1.721 | 3.159 | 2.26 | 3.766 | 2.86 | 0.2943 | 1.501 | 0.9503 | 0.7532 | 3.102 | 3.137 | 1.929 |
| **Evenness** | 0.05706 | 0.2427 | 0.09978 | 0.4363 | 0.1819 | 0.2616 | 0.068 | 0.0404 | 0.0442 | 0.2365 | 0.245 | 0.1434 |


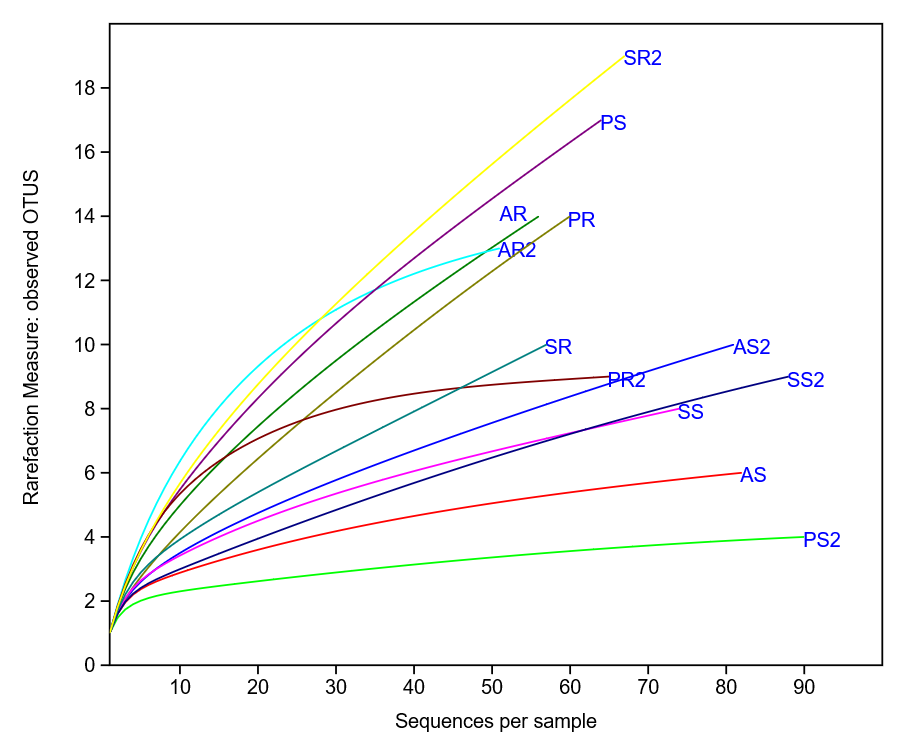


**Supplementary Fig. S2** The rarefaction curve for the endosphere microbial community of root and shoot of black rice at young and mature stage of plant in three variety of black rice (Amubi, Poreiton and Sempak). [AR- Young *Amubi* root, AR2-Mature *Amubi* root; AS- Young *Amubi* shoot, AS2- Mature *Amubi* shoot; PR- Young *Poreiton* root, PR2- Mature *Poreiton* root, PS- Young *Poreiton* shoot, PS2- Mature *Poreiton* shoot; SR- Young *Sempak* root, SR2- Mature *Sempak* root, SS- Young *Sempak* shoot, SS2- Mature *Sempak* shoot].

**Supplementary Table 3**- Correlation value (positive and negative) among the bacterial genera

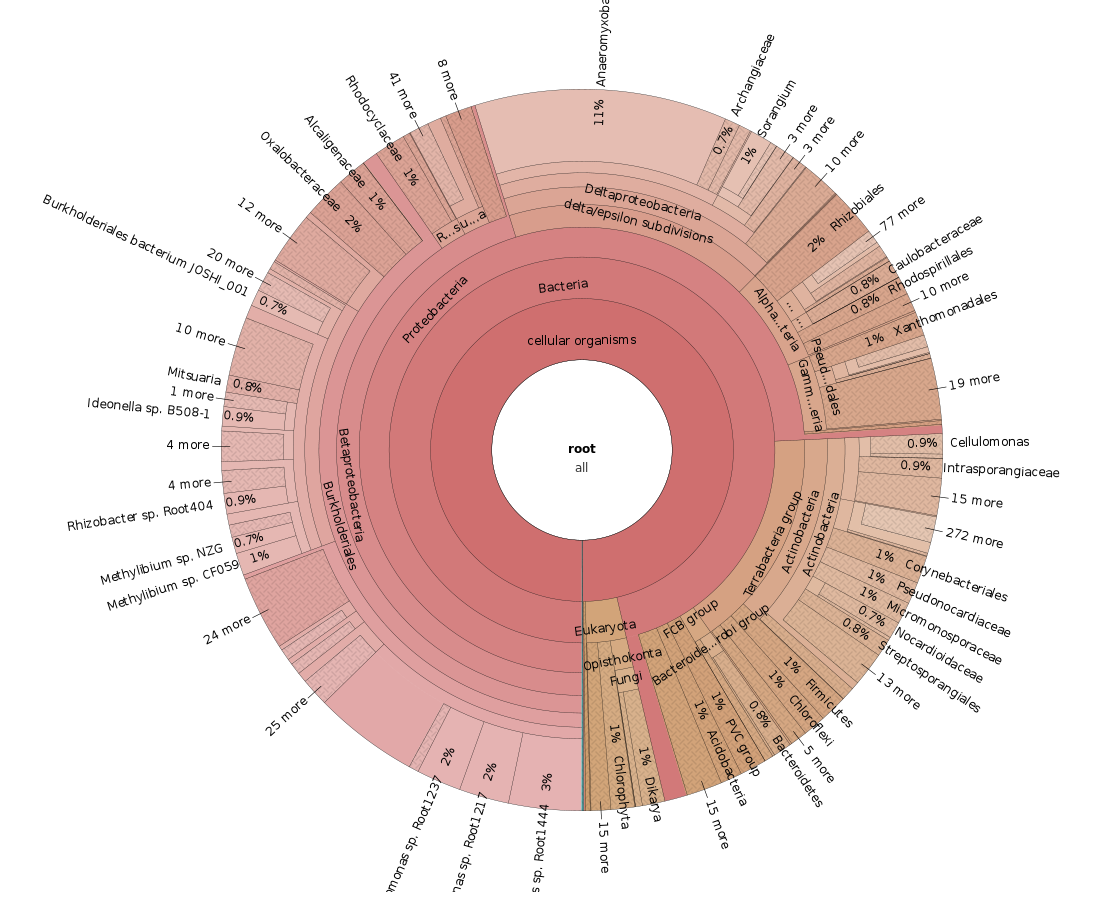


**Supplementary Fig. S3** Krona graph showing the Proteobacteria as major phyla at young stage


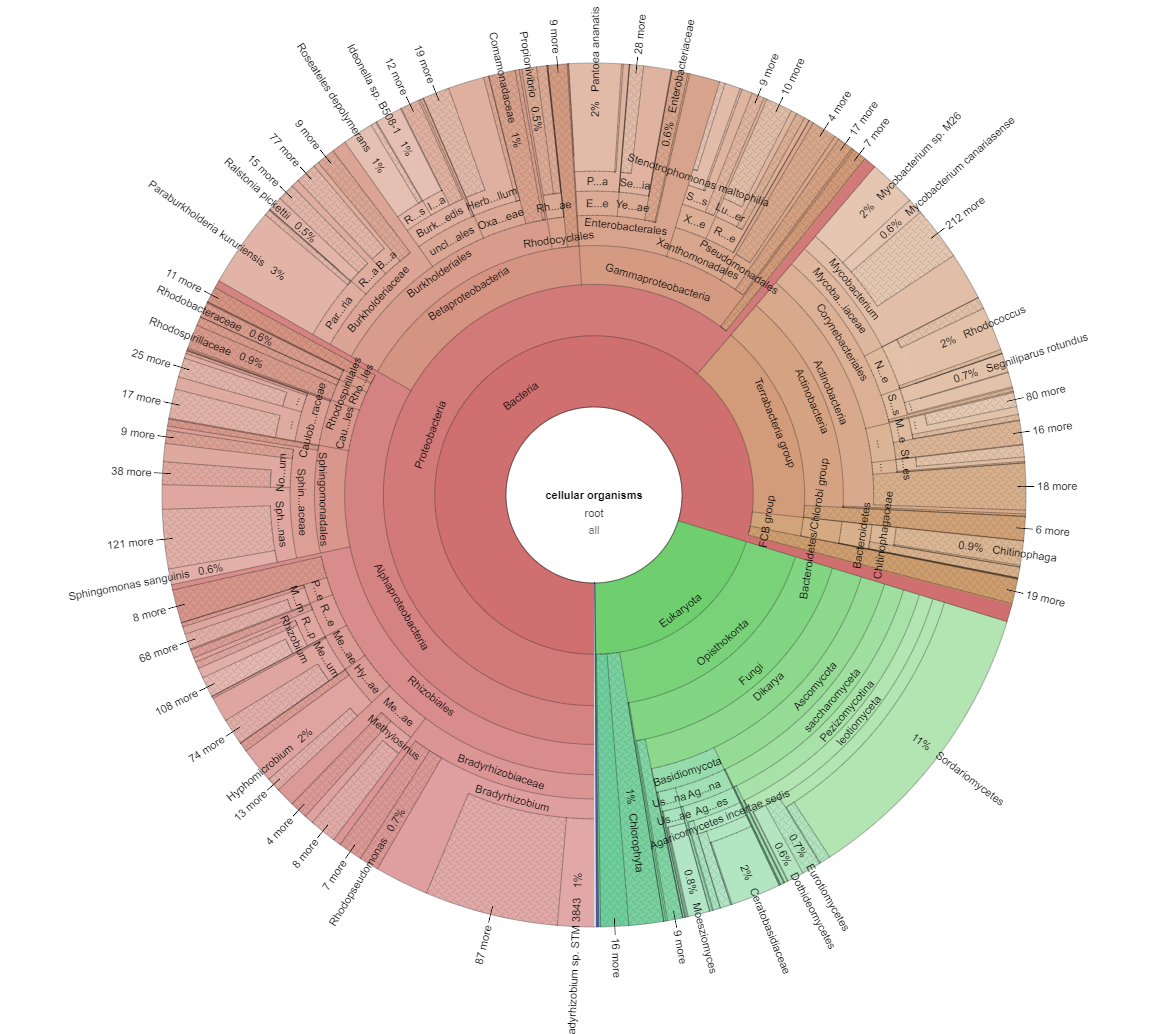


**Supplementary Fig. S4**  Krona graph showing the Proteobacteria as major phyla at mature stage

**Supplementary Table 4**. Antioxidant activity^*^ (TPC, TFC, AOA & FRSA) of root and shoot of three varieties of black rice at young and mature stage

|  | **TPC** | **TFC** | **AOA** | **FRSA** |
| --- | --- | --- | --- | --- |
| **AS** (a) | **41.45±1.06**  b, c, d, e, f, g, h, i | **18.47±1.27**  a,b,c,d,f,g,h,j,k,l | **25.79±0.96**  a,b,c,d,e,f,g,h,j,k,l | **0.36±0.05** |
| **AR** (b) | **30.60±0.64**  a, d, e, g, h, j, k, l | **9.43±0.70**  a,b,c,d,e,g,h,I,j,k,l | **7.62±0.53**  a,b,c,d,e,g,h,i,j,k,l | **1.10±0.10 e,f** |
| **AS2** (c) | **33.20±1.41**  a, d, e, g, h, k, l | **5.24±0.83**  a,b,e,I,j | **118.49±1.13** a,b,c,d,e,f,g,h,i,j,k,l | **0.14±0.02** |
| **AR2** (d) | **77.08±1.53**  a, b, c, e, f, h, I, j, l | **1.75±0.24**  a,b,e,f,I,j | **66.18±1.36**  a,b,c,d,e,f,g,h,i,j,k,l | **0.72±0.06** |
| **PS** (e) | **46.83±0.72**  a, b, c, d, e, f, g, h, I, j, k, l | **16.78±0.71**  b,c,d,e,f,g,h,I,k,l | **32.75±0.60**  a,b,c,d,e,f,g,h,i,j,k,l | **1.33±0.58** c |
| **PR** (f) | **33.9±1.00**  a, d, e, g, h, k, l | **5.77±0.58**  a,d,e,f,g,I,j,k | **5.79±0.66**  a,c,d,e,f,g,h,i,j,k,l | **1.33±0.22** c |
| **PS2** (g) | **52.60±0.72**  a, b, c, d, e, f, g, h, I, j, k, l | **3.94±0.54**  a,b,e,g,j,k | **83.62±0.46**  a,b,c,d,e,f,g,h,j,k,l | **0.42±0.18** |
| **PR2** (h) | **80.33±0.88**  a,b,c,e,f,g,h,I,j,k,l | **4.44±0.63**  a,c,e,f,h | **107.45±2.24** a,b,c,d,e,f,g,h,i,j,k,l | **0.37±0.04** |
| **SS** (i) | **35.41±1.32**  a,d,e,g,h,k,l | **20.95±1.02** b,c,d,e,f,g,h,I,j,k,l | **22.10±0.93**  b,c,d,e,f,g,h,i,j,k,l | **0.39±0.20** |
| **SR** (j) | **36.79±0.74**  a,d,e,g,h,k,l | **14.65±0.78** a,b,c,d,f,g,h,I,j,k,l | **16.39±1.03**  a,b,c,d,e,f,g,h,i,j,k,l | **0.64±0.07** |
| **SS2** (k) | **71.93±0.95**  a,b,c,e,f,g,h,I,j,k,l | **2.06±0.28**  a,c,e,f,I,j,k | **81.13±1.04**  a,b,c,d,e,f,g,h,i,j,k | **0.43±0.07** |
| **SR2** (l) | **90.49±0.90**  a,b,c,d,e,f,g,h,I,j,k,l | **2.18±0.34**  a,c,e,I,j,l | **80.42±0.52**  a,b,c,d,e,f,h,I,j,l | **0.42±0.58** |

Values given are mean of three replicates, ± significance error, values followed by the letters were significantly different (Tuckey Test*;*P < 0.05).

**Supplementary table 5**- Correlation value (positive and negative) between antioxidants and bacterial genera

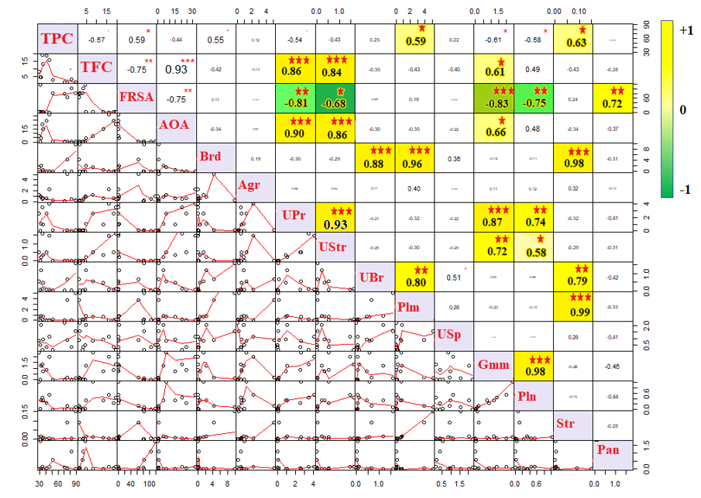


**Supplementary Fig. S5** Antioxant assay in three variety of black scented rice. Graphs show mean±SE. [AR- Young *Amubi* root, AR2-Mature *Amubi* root; AS- Young *Amubi* shoot, AS2- Mature *Amubi* shoot; PR- Young *Poreiton* root, PR2- Mature *Poreiton* root, PS- Young *Poreiton* shoot, PS2- Mature *Poreiton* shoot; SR- Young *Sempak* root, SR2- Mature *Sempak* root, SS- Young *Sempak* shoot, SS2- Mature *Sempak* shoot]. (**B)** Correlation of antioxidant activity with the bacterial genera. Significance levels P < 0.05, P < 0.01 and P < 0.001 are indicated by *, ** and *** respectively.


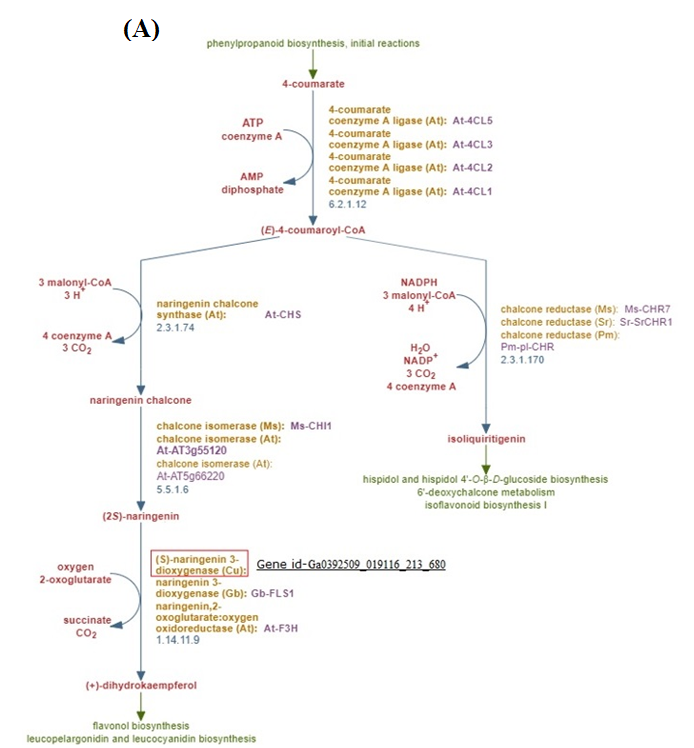


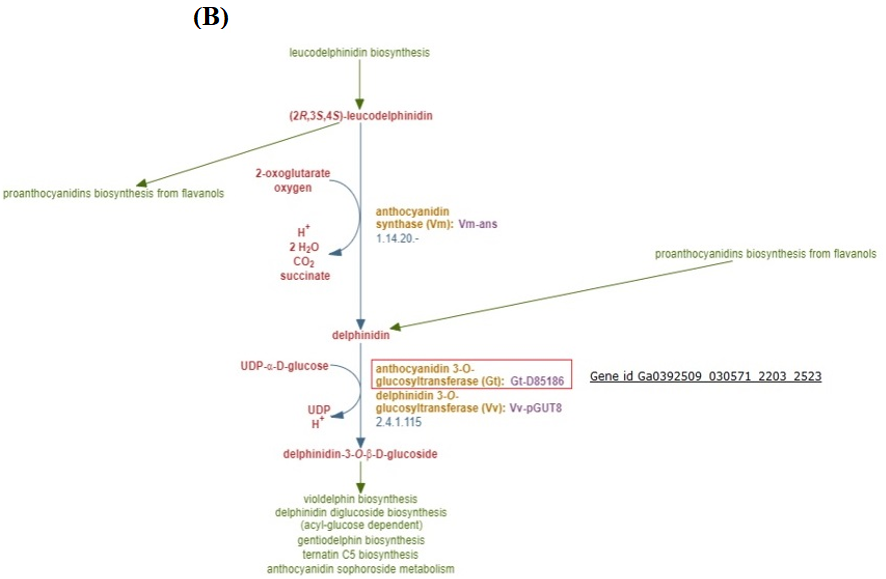


- **Supplementary Fig. S6**  Pathway involved in (**A)** Role of naringenin-3-dioxygenase in Flavonol biosynthesis^29^ (**B)** Role of anthocyanidin 3-O-glucosyltransferase in Anthocyanin biosynthesis pathway^29^ as identified in the endophytic functional microbiome of scented black rice. Genes inside boxes represent the same gene obtained in endophytic microbiome of scented black rice.

**Supplementary R script. R1---**

**R codes for correlation within the bacterial genera**

> library(Hmisc)

> getwd()

> setwd("C:/Users/HP/Desktop/")

> A1 <- read.csv("data.csv")

> A1

> r.names <- A1[,1]

> r.names

> mat_A1 <- data.matrix(A1[,2:ncol(A1)])

> res <- cor(mat_A1)

> rcorr(res,type=c("spearman"))

> res2$r

> res2 <- rcorr(as.matrix(res))

> res2$r

> res2$P

> library(corrplot)

> corrplot(res2$r, type="upper", order="hclust", p.mat=res2$P, sig.level=0.05, insig="blank")

**Supplementary R script. R2---**

**R codes for correlation within the bacterial genera and antioxidant activity**

library(PerformanceAnalytics)

##data=cv

cv <- read.csv(file.choose(), header = TRUE, sep = ",") # attach malabika_correlation.csv

chart.Correlation(cv, histogram=TRUE, pch=19, method = "pearson")

**Supplementary R script R3---**

**R codes for bubble diagram**

library(ggplot2)

dd <- read.csv(file.choose(), header = T, sep = ",")

head(dd)

ggplot(dd, aes(x = Variable, y = gene)) +

geom_point(aes(color = Variable, size = data), alpha = 0.5) +

scale_size(range = c(0.5, 12)) + labs(x = "Variable", y = "Bacteria sp.") + theme_minimal()

**Supplementary table 6** Statistically significant (t-test P < 0.05) genes classified under hierarchical KEGG orthology at young and mature stage of black rice plant. Abundance of each gene at a given time point is given as well as the taxonomic alignment based on BLAST.
